# Determinants of the range of drugs prescribed in general practice: a cross-sectional analysis

**DOI:** 10.1186/1472-6963-7-132

**Published:** 2007-08-22

**Authors:** Dinny H de Bakker, Dayline SV Coffie, Eibert R Heerdink, Liset van Dijk, Peter P Groenewegen

**Affiliations:** 1 NIVEL Netherlands institute for health services research, PO Box 1568, Utrecht, The Netherlands; 2 Utrecht University, Department of Pharmacoepidemiology and Pharmacotherapy, Utrecht, The Netherlands; 3 Utrecht University, Department of Human Geography and Department of Sociology, Utrecht, The Netherlands

## Abstract

**Background:**

Current health policies assume that prescribing is more efficient and rational when general practitioners (GPs) work with a formulary or restricted drugs lists and thus with a limited range of drugs. Therefore we studied determinants of the range of drugs prescribed by general practitioners, distinguishing general GP-characteristics, characteristics of the practice setting, characteristics of the patient population and information sources used by GPs.

**Methods:**

Secondary analysis was carried out on data from the Second Dutch Survey in General Practice. Data were available for 138 GPs working in 93 practices.

ATC-coded prescription data from electronic medical records, census data and data from GP/practice questionnaires were analyzed with multilevel techniques.

**Results:**

The average GP writes prescriptions for 233 different drugs, i.e. 30% of the available drugs on the market within one year. There is considerable variation between ATC main groups and subgroups and between GPs. GPs with larger patient lists, GPs with higher prescribing volumes and GPs who frequently receive representatives from the pharmaceutical industry have a broader range when controlled for other variables.

**Conclusion:**

The range of drugs prescribed is a useful instrument for analysing GPs' prescribing behaviour. It shows both variation between GPs and between therapeutic groups. Statistically significant relationships found were in line with the hypotheses formulated, like the one concerning the influence of the industry. Further research should be done into the relationship between the range and quality of prescribing and the reasons why some GPs prescribe a greater number of different drugs than others.

## Background

Prescribing a drug is one of the most frequent therapeutical decisions by general practitioners (GPs). There are numerous different drugs on the market, which means that GPs usually have a choice of several different drugs even when treating the same health problem. The aggregated costs for prescription drugs are high and increasing, however, and several policy initiatives have therefore been implemented against the background of this spiralling expense. One such initiative is to restrict GP prescribing to drugs that are on an indicative drug list, which was introduced in the UK.

In the absence of an indicative drug list in the Netherlands, it is important to know what influences the range that GPs prescribe. In the Netherlands, 75 percent of all drugs are prescribed by GPs and 65% of consultations end with a prescription [1]. Moreover, drug costs are rising sharply and they constitute an important expense in health care [2]. Current health policies assume that prescribing is more efficient and rational when GPs work with formulary or restricted drugs lists and thus with a limited range of drugs [3,4]. An advantage of using a limited range of drugs is that GPs become more familiar with drug dosages, contra-indications, interactions, etc., resulting in a lower incidence of side effects and dangerous interactions [5,6]. Especially, the number of analogue drugs (drugs consisting of various chemical substances, but used for the same condition) should be limited [6]. This should translate into lower costs [7].

There have been previous studies of the prescribing patterns of GPs, but most studies focused on differences in the total amount of prescriptions or on prescriptions for a particular group of drugs [8-10]. Little is known to date about the variation in the numbers of different drugs (the range) that GPs prescribe and about the factors that might explain these variations between GPs. Two studies showed that the number of doctors in the practice is positively related to the range of drugs prescribed [6,11]. The last study also found higher ranges in practices with high patient lists per doctor, a high percentage of elderly and a heavy workload. One study found no consistent relationship between the adoption of new drugs and previous prescribing of drugs belonging to the same therapeutic class [12]. Taylor e.a. linked GP characteristics (including their information sources) to prescribing in terms of adding new drugs to their repertory [13]. Another study showed that for two out of six therapeutic groups there was a positive association between number of years in practice and range of drugs [14].

The purpose of this study is to determine whether there is variation between GPs in the range of drugs they prescribe and whether there is variation within therapeutic groups of drugs, and how these differences can be explained. Our analysis is at the level of GPs where most recent studies were at practice level. Secondly, more determinants were taken into consideration, especially with respect to the GPs' information sources and the influence of patient characteristics.

### Hypotheses

It can be expected that known determinants of rational prescribing are also relevant in explaining the variation in the range of drugs prescribed. These determinants partly relate to individual GPs' prescribing decisions [15,16]. Differences in the range of drugs prescribed will also depend on individual GP characteristics, the practice setting in which he works and the composition of the practice population [17]. Further the way GPs acquire knowledge about new drugs might play a role [18].

First, *individual characteristics of GPs *might influence the range. Gender of the GP is not assumed to be directly related to the range of drugs prescribed. The age of the GP might have an influence in so far as age indicates years of experience. The direction of the relationship is difficult to hypothesize beforehand, however, because age (more experience) might be related to a smaller range of drugs (as a consequence of less uncertainty) or to a broader range (as a consequence of less up-to-date knowledge on pharmacotherapy). The individual list size might be positively related to the range of drugs simply because a higher number of patients increases the chance on a wider range of drugs. Further we can assume that GPs that are more inclined to prescribe in general might also be inclined to prescribe more different drugs.

Second, the *practice setting *in which the GP works might be of influence. Previous research showed that the number of doctors in the practice was positively related to the range at practice level [6,11], which could be explained by the fact that the combined repertory of more doctors will generally be larger than those of individual doctors. This does not mean, however, that individual GPs within group practices have larger ranges, although the fact that patients tend to switch between GPs in a group practice might influence the range positively. Dispensing practices might have a smaller range of drugs (as a consequence of more pharmaco-therapeutic knowledge, or for simple economic reasons of wanting to restrict the number of drugs in stock), but data for dispensing practices might also contain drugs that were prescribed by hospital consultants, which would result in a broader range.

Third, the *practice population *might influence the range of drugs prescribed. In general a more complex practice population is hypothesized to be related to a broader range of drugs prescribed, as a consequence of greater use of health services, worse health and more diverse health problems of urban residents, especially where mental health is concerned. So we expect a broader range in urban areas, especially in deprived areas, in practices with a high percentage of elderly and patients from non-western origin [16,19,20]. More people with higher education and fewer publicly insured people in a practice might be related to a broader range (the higher educated may be more demanding and new drugs are not always reimbursable for publicly insured patients).

Finally the *GPs' pharmaceutical information sources *especially on new drugs might play a role. In general it can be assumed that GPs who use independent sources of information will prescribe a smaller range of drugs, because independent sources will stress the 'me too' character of many new drugs[3,9,15,21]. Collaboration between GP and pharmacist can lead to more restricted formulary based prescribing [22,23]. Reliance on the pharmaceutical industry as a source of information is assumed to be related to a broader range of drugs prescribed, because these sources will stress the advantages of new drugs [3,9,21,24-26].

## Methods

Data were analyzed from the Second Dutch National Survey of General Practice, a large scale study held in 2001. Data were collected in 104 computerized general practices with 195 GPs serving a population of approximately 400,000 residents. The data include background information on the patients involved, collected from the practice computer (list size, insurance type) and via a census (e.g. education, ethnicity, occupation), approximately 14,000 health interview surveys, and 1.4 million electronically coded medical records (consultations, diagnosis, prescriptions, referrals) kept of all patients contacting the GP in 2000/1 [27]. Further information on GP and practice characteristics were collected with GP/practice questionnaires. The GP questionnaire included factors affecting GP prescribing behaviour. The Second Dutch National Survey of General Practice was carried out according to Dutch legislation on privacy. The privacy regulation of the study was approved by the Dutch Data Protection Authority.

Prescription data that could be traced to individual GPs were selected from the electronic medical records. Only GPs with more than 500 prescribed medications were included. This resulted in data for 138 GPs working in 93 practices.

Ninety-two percent of all medication prescribed was classified by active substance according to the Anatomical Therapeutical Chemical (ATC) classification code developed by the World Health Organisation (WHO) [28].

### Measurement of the range (dependent variable)

We define the range as the number of different drugs prescribed within a certain ATC-system main group (ATC 3) (Table 1), thereby focusing on the number of analogue drugs [6]. Not all the therapeutic groups within the ATC-system were relevant for this study. Some therapeutic groups were excluded for reasons that included the following:

**Table 1:** Mean and variation between GPs in range of the selected therapeutical main or subgroups*

	ATC	Average range	Coefficient of variation	Min	Max	Number of drugs on the market	Range as % of drugs on the market
Antacids, drugs for treatment of peptic ulcers/flatulence	A02	10.90	28.85	3	20	32	34.1
Laxatives	A06	7.41	32.60	2	13	24	30.9
Drugs used in diabetes	A10	8.80	26.02	3	14	17	51.8
Insulins	A10A	3.78	38.15	1	7	7	54.0
Oral blood glucose lowering drug	A10B	5.12	22.56	2	7	10	51.2
Antithrombotic agents	B01	5.95	26.27	1	10	27	22.0
Cardiac therapy	C01	6.06	26.06	1	10	32	18.9
Diuretics	C03	7.04	20.04	3	9	15	46.9
Beta blocking agents	C07	8.31	26.56	4	15	26	32.0
Agents acting on the renin-angiotensin system	C09	12.09	31.30	3	22	31	39.0
Antifungals for dermatological use	D01	6.89	26.69	2	12	17	40.5
Corticosteriods, dermatological preparations	D07	12.43	25.40	5	22	27	46.0
Sex hormones and modulators of the genital system	G03	21.70	24.78	9	36	56	38.8
Hormonal contraceptives for systemic use	G03A	9.30	22.71	4	14	17	54.7
Estrogens	G03C	2.89	24.98	1	5	6	48.2
Rest of G03 (G03REST)		9.51	37.70	2	19	33	28.8
Antibacterials for systemic use	J01	17.18	15.98	10	24	79	21.7
Anti-inflammatory and antirheumatic products	M01	9.02	26.04	4	17	25	36.1
Anti-inflammatory/-rheumatic products. non-steriods	M01A	8.92	25.53	4	17	22	40.5
Analgesics	N02	12.62	23.49	4	21	40	31.6
Opioids	N02A	4.33	28.91	1	7	17	25.5
Other analgesics and antipyretics	N02B	3.80	23.55	1	6	13	29.2
Antimigraine preparations	N02C	4.48	38.55	1	9	10	44.8
Psycholeptics	N05	21.70	27.61	6	37	76	28.6
Antipsychotics	N05A	7.85	45.58	1	22	31	25.3
Anxiolytics	N05B	7.19	27.84	2	12	16	44.9
Hypnotics and sedatives	N05C	6.83	25.36	2	11	29	23.6
Psychoanaleptics	N06	12.48	24.26	4	19	33	37.8
Antidepressants	N06A	11.24	23.32	4	17	24	46.8
Nasal preparations	R01	7.92	24.17	3	12	17	46.6
Anti-asthmatics	R03	12.86	20.55	6	20	26	49.5
Cough and cold preparations	R05	4.34	36.89	1	9	22	19.7
Antihistamines for systemic use	R06	10.20	27.96	3	17	25	40.8
Ophthalmologicals	S01	16.98	41.17	5	39	79	21.5
							
Overall Range		232.87	20.61	111	353	755	30.8

*compared to the number of drugs on the market in 2001.

- they offered too little drug choice; only a few drugs are available within these groups (e.g. N07: other CNS drugs, including parasympathomimetics)

- they concerned an unusual disorder which has a very low prevalence (e.g. H: systemic hormonal preparations excluding sex hormones)

- they concerned diseases which are treated only by specialists and not by GPs (e.g. L: antineoplastic and immunosuppressive drugs)

On the other hand, there were some important subgroups (ATC-4) for which it would be favourable to determine the range for these groups separately, e.g. the anti-psychotics (N05A), the anti-depressants (N06A), etc.

ATC codes were recorded at GP level. The explanatory analysis used an overall score for the range, instead of separate range values for the different therapeutic groups. This score was computed by summing the ranges over the ATC3/ATC4 subgroups (see total in table 1). Factor analysis showed that the scores for the various subgroups were unidimensional.

### Measurement of the explanatory variables

The following explanatory variables were included in the analysis. Unless otherwise indicated, these were derived from the GP/practice questionnaires.

#### GP background characteristics

- Age and gender of the GP.

- Individual list size (practice computer).

#### Practice characteristics

- Type of practice (single-handed, duo, group; entered as dummy variables in the analysis),

- dispensing practice,

- number of prescriptions per listed patient per year (electronic medical records/practice computer): we hypothesized a relationship at GP-level. Because of data problems we had to include this variable at practice level, however.

#### Practice population characteristics

- Degree of *urbanization *(as classified by Statistics Netherlands on a five-point scale ranging from not urbanized to very strongly urbanized; entered as dummy variables in the analysis),

- practice located in deprived urban area (as classified for additional remuneration of GPs [27]),

- % of practice population older than 75 years (practice computer),

- % of practice population with higher education, according to definition Statistics Netherlands (census),

- % of practice population publicly insured (practice computer),

- % of practice population of non-western origin according to definition (census).

#### GPs' pharmaceutical information sources

- Participation in postgraduate courses (hours per year),

- participation in Pharmaco-therapeutic Audit Meetings (frequency per year),

- number of different written sources (max. 4) used for information on new drugs,

- number of different oral sources (max. 3) used for information on new drugs,

- number of representatives of pharmaceutical industry received in the last four weeks,

- mean frequency of use of independent information sources like guidelines and formularies (4 item five-point scale ranging from never to often, α = .64),

- mean frequency of use of information sources in the pharmaceutical industry (on a five-point scale ranging from never to often, α = .65).

The scales on frequency of use of independent information and information from industry appeared to be independent factors in a factor analysis with all information items [21].

### Analysis

Descriptive information is given for the range per ATC group. The coefficient of variation (equal to (standard deviation/mean)*100) is given instead of the standard deviation because this enables comparison between ATC-groups. Bivariate (Pearson) correlation coefficients were computed between the explanatory variables and with the overall range. Multiple regression analysis was used to examine the joint relationship between the dependent variable and the explanatory variables. Hypotheses were tested one-tailed with a significance of p < 0.05, where we had a directional hypotheses and two-tailed when there was no directional hypotheses. The regression analysis was performed using multilevel analysis [30,31], due to the dependence of the observations for GPs working in the same practice.

## Results

Table 1 shows the ranges for the therapeutic main groups and subgroups selected. The range is of course dependent on the number of drugs on the market and the number of drugs on the market was therefore placed as a reference category, while the range was also computed as a percentage of the number of drugs on the market. We see clear variation between different therapeutic groups in the percentages of available drugs used. In the case of cardiac therapy (C01), antibacterials (J01), antithrombotic agents (B01) and hypnotics and sedatives (N05C), the average GP uses less than 25% of the available drugs on the market. In contrast, more than 50% of all available insulins (A10A), hormonal contraceptives (G03A), drugs used in diabetes (A10) and oral blood glucose lowering drugs (A10B) are used by the average GP. If we look at all therapeutic main groups together, the average GP uses 233 different drugs or 31% of the drugs available on the market.

The second type of variation we can perceive in the table is between GPs. The total number of drugs prescribed varies between 111 and 353, or between 15% and 47% of the available drugs on the market. There are differences between therapeutic main groups and subgroups in this respect, the coefficient of variation ranging from 16 for antibacterials to 46 for antipsychotics. The fact that for both these drug groups the range as a percentage of available drugs used was low shows that a low range as a percentage of available drugs used does not imply automatically little variation between GPs.

Descriptive information of the explanatory variables used has been given in table 2, which also shows the relationship of these variables with the overall range. The range is statistically significantly larger in non-urbanized areas, in dispensing practices, for GPs with larger patient lists, in practices with more patients with higher education, in practices that frequently received representatives of the pharmaceutical industry and more frequently used information sources of the industry, and in practices with a high prescription volume per patient. We see a statistically significant smaller range for GPs working in deprived urban areas. Table 3 shows the statistically significant correlation coefficients between the explanatory variables. The correlations are moderate at most, which means that they can be entered together in a multivariate analysis.

**Table 2:** Independent variables: descriptive characteristics and bivariate correlations with overall range, tested one-tailed*

	mean/%	Sd	corr. with range
Age GP (mean/sd)^a^	47.7	6.1	0.05
Gender GP (% female)^a^	20		-0.09
*Type of practice*			
Single-handed (%)	35		0.07
duo practice (%)	25		0.13
Group practice (%)	40		-0.04
*Degree of urbanisation*			
very strong (%)	17		-0.15
Strong (%)	23		0.00
moderate (%)	18		-0.04
little (%)	30		0.01
not (%)	12		0.20*
Deprived urban area (%)	9		-0.17*
Dispensing practice (%)**	10		0.25*
Individual list size (mean/sd)	2193	560	0.26*
% patients > 75 yrs (mean/sd)	6.1	2.8	0.07
% patients with higher education (mean/sd)	23.1	11.5	0.22*
% patients publicly insured (mean/sd)	66.9	8.7	0.12
% patients of non-western origin (mean/sd)	6.5	11.8	-0.01
Hours per year postgraduate courses (mean/sd)	55.4	62.2	-0.05
Participation per year in pharm. meetings (mean/sd)	7.5	4.1	0.00
Use written information sources (mean/sd)	2.1	1.2	0.13
Use oral information sources (mean/sd)	1.7	1.1	-0.10
Visits (last 4 weeks) representatives pharm. ind. (mean/sd)	2	2.9	0.18*
Use independent information sources (mean/sd)	3.9	0.8	-0.10
Use information sources industry (mean/sd)	1.4	0.6	0.15*
Prescriptions per patient per year (mean/sd)	6.5	11.8	0.38*

* p < 0.05.

** Where there was no directional hypothesis two-tailed testing was applied

**Table 3:** Pearson correlation coefficients between explanatory variables (only significant correlations. p < 0.05)

		1	2	3	4	5	6	7	8	9	10	11	12	13	14	15	16	17	18	19	20	21	22	23	24
1	age GP																								
2	female GP	-0.31																							
3	single handed		-0.17																						
4	duo practice			-0.42																					
5	group practice		0.27	-0.60	-0.47																				
6	very strong urban			0.24		-0.17																			
7	strong urban						-0.25																		
8	moderate urban						-0.21	-0.26																	
9	little urban			-0.28		0.31	-0.29	-0.36	-0.31																
10	not urban							-0.20	-0.17	-0.24															
11	deprived urban area		0.28				0.59			-0.21															
12	dispensing							-0.19			0.40														
13	individual list size		-0.37	0.36		-0.37			0.25			-0.26													
14	% > 75 yrs		-0.24			-0.27				-0.18															
15	% higher education						0.36	0.20		-0.24	-0.21	0.54	-0.19												
16	% sick fund insured															-0.60									
17	% non-western		0.20	0.26		-0.18	0.52			-0.23		0.36					0.45								
18	postgraduate courses						0.17								0.19										
19	part. pharm. meetings																								
20	written info sources					-0.17								0.17											
21	oral info sources				-0.17	0.21	0.20						-0.24												
22	visits repres. ind.		-0.21	0.39		-0.34			0.35	-0.28				0.38							0.31				
23	use indep. sources									0.24				-0.20						0.19			-0.29		
24	use sources ind.			0.22																	0.29		0.41		
25	prescriptions per patient						-0.18				0.55		0.43	-0.18	0.29	-0.35	0.24								

The results of the multilevel analysis are shown in table 4. In the empty model (without explanatory variables), we found that the amount of variation at GP-level differed significantly from zero. There was no significant variation at practice level.

**Table 4:** Multilevel regression analysis of the explanatory variables on the overall range of different medicines prescribed by GPs in 2001

	Empty model	Full model	
	Estimate (standard error)	Estimate (standard error)	Hypothesis
practice level fixed effects			
- prescriptions per patient		0.2583 (0.1005)*	Confirmed
			
GP level fixed effects:			
- visits representatives industry		0.9289 (0.0456)*	Confirmed
- individual list size		0.0000765(0.0002074)*	Confirmed
			
variance components:			
practice level intercept	0.1276(0.1521)	0.2684(0.1424)	
GP level intercept	0.8419(0.1749)*	0.5914(0.1324)*	
			
intraclass correlation	13.2%	31.2%	

*p < 0. 05, one-tailed; N(practices) = 93; n(GPs) = 138.

The clustering of GPs within practices – the intraclass correlation – is relatively strong, which means that the ranges for GPs in the same practices are correlated (0.312 in the full model). Table 4 shows that the prescribing volume at practice level was significantly and positively related to the overall range, which also applied at GP level to the individual list size and the frequency of visits of representatives of the pharmaceutical industry at practice level. All of this confirms our hypotheses.

Other hypotheses were not confirmed. We did not find that urban location is related to a broader range, nor did we find wider ranges in group practices, in practices with a high proportion of elderly patients, a high proportion of people with higher education or a low proportion of publicly insured people. In the field of pharmaceutical knowledge, we did not find significant relationships with access to independent information sources or participation in Pharmaco-Therapeutic Audit Meetings. Multiple regression analysis showed that 38% of the total variance could be explained by the variables used.

## Discussion

In this article, we analyzed the concept of the range of drugs prescribed as an alternative or supplement to more usual measurements like the total number of prescriptions. The material in this article shows this to be a useful addition, especially when more precise clinical indicators are absent. Firstly because the range shows clear variations between ATC main groups and subgroups, and between GPs; secondly because it was possible to explain the variation between GPs by factors that have a theoretically interpretable relationship to the range. GPs with larger patient lists, GPs with higher prescribing volumes and GPs who frequently receive representatives from the pharmaceutical industry turned out to have a broader range when controlled for other variables. These statistical relationships could be linked to the hypotheses we formulated on the basis of the literature or theoretical considerations.

A measure which is comparable to the range is DU90% (the number of drugs constituting 90% of the volume expressed in DDDs). This has been used for GP data in Stockholm, Sweden, as a general quality indicator in feedback [32,33]. In an intervention project aimed at decreasing the number of inhabitants per GP, no systematic change in DU90% could be detected [33].

For measures of prescribing variation, ideally, guideline recommendations should be taken into account. Bergman e.a. [32] did not find a relationship between DU90% and adherence to a local formulary. The theoretical link between the range of drugs prescribed and quality of drug prescribing is based on the use of restrictive formularies or guidelines. This link was not studied in our article. It would require specific information on the indication to prescribe and on the link between indications and guidelines or formularies. However, in the absence of detailed information on indications, the range of drugs could be used as an overall measure in addition to other drug-specific measures [34].

The relationship of the range of drugs prescribed with the number of prescriptions per patient and list size can be easily understood by the fact that the chance that a greater number of different drugs will be prescribed is higher when the number of prescriptions is higher. A second explanation concerns the role of specialist initiated prescription that is subsequently followed by repeat prescriptions by GPs, a well known phenomenon in the Netherlands. This might vary between GPs because GPs who prescribe more usually also refer more to hospital consultants [35]. Therefore high prescribing GPs might also prescribe more repeat prescriptions initiated by specialists, that often deviate from (GP-)guidelines. An additional explanation for the relationship with prescriptions per patient may be that a broader drug repertory increases the inclination to prescribe because it increases the therapeutic possibilities of the doctor. The causal direction is difficult to infer from cross-sectional data as used by us. This is, of course, a limitation of this study.

A further limitation of our research is that the possibility of multicollinearity cannot completely be ruled out. Although the correlations between the independent variables are below the threshold level of .60 the correlations with non-urbanized areas (.55) and dispensing practice (.43) come close to the threshold. In dispensing practices prescriptions of GPs dispensed by the practice cannot be separated from specialist prescriptions. Removing the 14 dispensing GPs from the analysis lowered the coefficients a bit but did not change our overall conclusion.

GPs working in partnerships did not appear to have a broader range when controlled for other variables. This seems surprising, because we know from previous research that GPs in group practices have a broader range. We expected to see this also on individual level since their patients more often switch between GPs in group practices. Table 3 shows that GPs in group practice more often use oral information sources and receive fewer representatives of the pharmaceutical industry. On the other hand, working in a group practice is often associated with a stronger orientation to esteem by colleagues and to professional guidelines, leading to more rational prescription behaviour and therefore to a lower range[36,37]. The more frequent use of oral information (which will often be from colleagues) may point to this (see table 3). This might neutralize the range-broadening effects of patients switching between doctors.

Contrary to our expectations, we did not find any statistical relationship with factors associated with the composition of the practice or with the closely linked factor of the location of the practice. These were, in fact, proxy-indicators for differences in morbidity. This brings us to the second limitation of the study, which is that we did not take the diagnosis underlying the prescriptions into account. As a follow-up to this study, we recommend an analysis of the relationship between range and rational prescribing, taking diagnosis and comorbidity into account. This would also provide better opportunities to link the range of drugs prescribed to guidelines. A more specific follow-up study would be to investigate how computerized decision support systems affect the range of drugs prescribed.

Further and more detailed research is necessary into the differences between therapeutic main groups and subgroups with regard to the percentages of available drugs prescribed. We would hypothesize that GPs have a greater inclination to switch to other drugs in therapeutic groups where side-effects play an important role (i.e. hormonal contraceptives). The same could be true of therapeutic drugs with limited or variable effectiveness (i.e. antifungals for dermatological use). This is the grey area where representatives of the pharmaceutical industry play a role, stressing the advantages of their products compared to other products, in the form of fewer side-effects and better results.

## Conclusion

We conclude that the range of drugs prescribed is a useful concept that could be used in addition to prescribing volume in research into prescription behaviour, especially when information concerning the indication is lacking. Restriction of the range of drugs as a policy instrument needs more thorough underpinning by means of further analysis of both quality and cost correlates of a smaller range of drugs prescribed.

## Competing interests

The author(s) declare that they have no competing interests.

## Authors' contributions

DDB participated in designing the study, did the final data analysis and drafted the final manuscript. DC prepared the data, did preliminary analysis and wrote the preliminary report. ERH participated in designing the study and helped in the interpretation of the data. LVD analyzed specifically the explanatory variables. PG helped in the design of the study and co-drafted the manuscript. All authors read and approved the final manuscript.

## Pre-publication history

The pre-publication history for this paper can be accessed here:


